# A Novel Strain of Fusarium oxysporum Alternavirus 1 Isolated from *Fusarium oxysporum* f. sp. *melonis* Strain T-BJ17 Confers Hypovirulence and Increases the Sensitivity of Its Host Fungus to Difenoconazole and Pydiflumetofen

**DOI:** 10.3390/v16060901

**Published:** 2024-06-02

**Authors:** Huihui Hua, Xinyi Zhang, Li Liu, Xuehong Wu

**Affiliations:** College of Plant Protection, China Agricultural University, Haidian District, Beijing 100193, Chinazxy18511290531@163.com (X.Z.); liulill1124@163.com (L.L.)

**Keywords:** *Fusarium oxysporum* f. sp. *melonis*, *Alternavirus*, Fusarium oxysporum alternavirus 1-FOM (FoAV1-FOM), hypovirulence, increased sensitivity, difenoconazole, pydiflumetofen

## Abstract

In the current study, a novel strain of Fusarium oxysporum alternavirus 1 (FoAV1) was identified from the *Fusarium oxysporum* f. sp. *melonis* (FOM) strain T-BJ17 and was designated as Fusarium oxysporum alternavirus 1-FOM (FoAV1-FOM). Its genome consists of four dsRNA segments of 3515 bp (dsRNA1), 2663 bp (dsRNA2), 2368 bp (dsRNA3), and 1776 bp (dsRNA4) in length. Open reading frame 1 (ORF1) in dsRNA1 was found to encode a putative RNA-dependent RNA polymerase (RdRp), whose amino acid sequence was 99.02% identical to that of its counterpart in FoAV1; while ORF2 in dsRNA2, ORF3 in dsRNA3, and ORF4 in dsRNA4 were all found to encode hypothetical proteins. Strain T-BJ17-VF, which was verified to FoAV1-FOM-free, was obtained using single-hyphal-tip culture combined with high-temperature treatment to eliminate FoAV1-FOM from strain T-BJ17. The colony growth rate, ability to produce spores, and virulence of strain T-BJ17 were significantly lower than those of T-BJ17-VF, while the dry weight of the mycelial biomass and the sensitivity to difenoconazole and pydiflumetofen of strain T-BJ17 were greater than those of T-BJ17-VF. FoAV1-FOM was capable of 100% vertical transmission via spores. To our knowledge, this is the first time that an alternavirus has infected FOM, and this is the first report of hypovirulence and increased sensitivity to difenoconazole and pydiflumetofen induced by FoAV1-FOM infection in FOM.

## 1. Introduction

*Fusarium oxysporum*, predominantly a phytopathogen, can infect a variety of plants and cause Fusarium wilt [1] or Fusarium root rot [2,3]. To date, there are seventeen mycoviruses with complete genomic sequences that have been reported to infect *F. oxysporum*. Three mycoviruses, Fusarium oxysporum f. sp. dianthi mycovirus 1 (FodV1) [4,5,6], Fusarium oxysporum f. sp. dianthi mitovirus 1 (FodMV1) [7], and Fusarium oxysporum f. sp. dianthi hypovirus 2 (FodHV2) [8], were recorded as infecting *F. oxysporum* f. sp. *dianthi*. Nine mycoviruses, Fusarium oxysporum f. sp. cubense ourmia-like virus 1 (FocOLV1), FocOLV2, FocOLV3, FocOLV4, Fusarium oxysporum f. sp. cubense mitovirus 1 (FocMV1), FocMV2, FocMV4, Fusarium oxysporum f. sp. cubense mymonavirus 1 (FocMyV1), and Fusarium oxysporum f. sp. cubense negative-stranded RNA virus 1 (FocNSRV1), were documented with the ability to infect *F. oxysporum* f. sp. *cubense* [9]. Fusarium oxysporum ourmia-like virus 1 (FoOuLV1) [10] and Fusarium oxysporum alternavirus 1 (FoAV1) [11] were isolated from *F. oxysporum*, causing Fusarium wilt of bitter gourd and Fusarium wilt of *Lilium brownii*, respectively. Fusarium oxysporum mitovirus 1 (FoMV1) [2] and Fusarium oxysporum mymonavirus 1 (FoMyV1) [3] were identified from *F. oxysporum* and incited Fusarium root rot of tobacco and Fusarium root rot of capsicum, respectively. Hadaka Virus 1 (HadV1) [12] was reported to infect *F. oxysporum*, causing diseases on tomato. However, no mycoviruses have been reported to be associated with *F. oxysporum* f. sp. *melonis* (FOM) until now.

Among the seventeen mycoviruses mentioned above, only one mycovirus (FoAV1) belongs to the family *Alternaviridae*, which consists of one genus, *Alternavirus.* Besides FoAV1, which was isolated from *F. oxysporum*, there have been seven other alternaviruses identified from seven species of *Fusarium*, namely Fusarium avenaceum alternavirus 1 (FaAV1), Fusarium graminearum alternavirus 1 (FgAV1), Fusarium incarnatum alternavirus 1 (FiAV1), Fusarium nanum alternavirus 1 (FnAV1), Fusarium poae alternavirus 1 (FpAV1), Fusarium pseudograminearum alternavirus 1 (FpgAV1), and Fusarium solani alternavirus 1 (FsAV1) [11,13,14,15,16,17,18,19], which infected *F*. *avenaceum*, *F*. *graminearum*, *F*. *incarnatum*, *F*. *nanum*, *F*. *poae*, *F*. *pseudograminearum*, and *F*. *solani*, respectively.

Three mycoviruses (FodV1, FoOuLV1, and FoMyV1) associated with *F. oxysporum* [3,4,10], four mycoviruses (Fusarium graminearum hypovirus 2, FgHV2; Fusarium graminearum virus-ch9, FgV-ch9; Fusarium graminearum virus 1, FgV1; Fusarium graminearum gemytripvirus 1, FgGMTV1) identified from *F. graminearum* [20,21,22,23], and one mycovirus (Fusarium pseudograminearum megabirnavirus 1, FpgMBV1) isolated from *F*. *equiseti* [23], are known to be capable of conferring hypovirulence on their host fungi. Additionally, FoAV1 could horizontally transmit from its host fungus (*F. oxysporum*, donor strain), causing Fusarium wilt of *L*. *brownii* to another strain of *F. oxysporum* (*F. oxysporum* f. sp. *Momordicae*, recipient strain, which causes Fusarium wilt of bitter gourd), and thus conferred hypovirulence on its recipient strain [11]. In particular, a strain of Fusarium poae virus 1 (FpV1-Fa) identified from *F. asiaticum* could transfer from its host fungus, *F. asiaticum*, to two other species of *Fusarium*, *F. poae* and *F. tricinctum*; consequently, the virulence of the FpV1-Fa recipient strain (*F. poae* or *F. tricinctum*) was reduced [24].

Difenoconazole is a demethylase inhibitor (DMI) fungicide and can control diseases caused by *Alternaria* [25,26], as well as Fusarium wilt [27,28,29,30] or Fusarium root rot [31] incited by *F. oxysporum*. Pydiflumetofen, as a succinate dehydrogenase inhibitor (SDHI) fungicide, can effectively control Fusarium wilt incited by *F. oxysporum* f. sp. *niveum* [32]. There were no reports that difenoconazole and pydiflumetofen were used to control muskmelon Fusarium wilt caused by FOM.

In the present study, the discovery of mycoviruses present in 148 strains of FOM was made by metatranscriptome sequencing, and a novel strain of FoAV1 was identified from strain T-BJ17 and named FoAV1-FOM. The effect of FoAV1-FOM infection on colony growth, spore production, mycelial biomass, virulence, and sensitivity to difenoconazole and pydiflumetofen from its host fungus were evaluated. Assays on the vertical transmission of FoAV1-FOM via spores were also conducted.

## 2. Materials and Methods

### 2.1. Fungal Strains

In total, 149 strains of *Fusarium* were used in this study. Among them, 148 strains (including T-BJ17) were originally isolated from diseased muskmelon roots with symptoms of Fusarium wilt that were collected from nine provinces (Fujian, Guangdong, Heilongjiang, Hunan, Jilin, Jiangxi, Liaoning, Shandong, and Zhejiang provinces), a municipality (Beijing municipality), and two autonomous regions (Inner Mongolia autonomous region and Ningxia Hui autonomous region) across China (Table S1), which were identified as FOM according to the methods described previously [33,34,35,36]. These 148 strains were used for metatranscriptome sequencing. T-BJ17-VF is an isogenic strain of T-BJ17 that was obtained by eliminating FoAV1-FOM from T-BJ17. All these *Fusarium* strains were grown on PDA plates in the dark at 25 °C for 7 d prior to use.

### 2.2. Extraction and Purification of RNA

The 148 strains of FOM used for metatranscriptome sequencing were divided into 30 groups (Table S1), and the mycelia of each strain in the same group were mixed equally. Total RNA from each group was extracted using TRIpure Reagent (Aidlab Biotechnologies, Beijing, China) according to the manufacturer’s instructions. The concentrations and quality of the RNA samples were evaluated, which were subsequently pooled by mixing 1 µg of RNA from each group to obtain one single sample with a final concentration (~200 ng/µL). The single sample was sent to the Shanghai Biotechnology Corporation (Shanghai, China) for metatranscriptome sequencing using an Illumina X-TEN instrument with paired-end program. The dsRNA extraction and purification of T-BJ17 was carried out as previously described [13,29]. Both dsRNA and total RNA samples were stored at –80 °C until further use.

### 2.3. Metatranscriptome Sequencing

The cDNA library was constructed through rRNA-depleted total RNA by using TruSeq Stranded Total RNA Library Prep Gold (Illumina, San Diego, CA, USA, 20020598). The library quality was checked using the Qubit^®^ 2.0 Fluorometer (Invitrogen, Carlsbad, CA, USA, Q32866) and Agilent Technologies 2100 Bioanalyzer (Agilent Technologies, Santa Clara, CA, USA). Clean reads were obtained by several filter steps (unqualified reads including low-quality scores (<20) in the raw data, adaptor sequences, reads of less than 20 bp, and host-mRNA and -rRNA sequences). These reads were de novo assembled into large contigs using the scaffolding contig algorithm. Subsequently, all the contigs obtained were compared with the National Center for the Biotechnology Information (NCBI) non-redundant (NR) database. BLASTx was used to determine the best virus matches of the mycovirus-like contigs.

### 2.4. Reverse Transcription–Polymerase Chain Reaction (RT-PCR)

To verify the presence of the putative mycoviruses, specific primers were designed based on the assembled contigs (Table S2). The complementary DNA (cDNA) of a single strain was synthesized using Reverse Transcriptase M-MLV (TaKaRa, Dalian, China) following the manufacturer’s protocol. The RT-PCR was performed with a 25 µL PCR mixture as Zhao et al. described [37]. The PCR products were verified by 1% agarose gel electrophoresis and sequenced by the Tsingke Biotechnology Co., Ltd. (Beijing, China). The amplification for each potential viral sequence was conducted three times, and the amplification products of these three repetitions were sequenced separately.

### 2.5. Synthesis and Molecular Cloning of Complementary DNA (cDNA)

The synthesis and molecular cloning of cDNA was performed as described previously [38,39]. The 5′- and 3′-terminal sequences of the mycovirus genome were obtained using the RNA-ligase-mediated rapid amplification of cDNA ends (RLM-RACE) method, which involved ligating each dsRNA with an adaptor PC3-T7 loop using T4 RNA ligase (TaKaRa, Dalian, China) according to the method described previously [39]. A PC2 primer and sequence-specific primers (Table S3) were used for the amplification of terminal sequences. Sequencing was performed by the Beijing Tianyihuiyuan Biotechnology Co., Ltd. (Beijing, China).

### 2.6. Analysis of Sequences and Phylogenetic Analysis

Putative open reading frames (ORFs) in the dsRNAs were identified using ORF Finder (https://www.ncbi.nlm.nih.gov/orffinder/, accessed on 7 February 2024) from the National Center for Biotechnology Information (NCBI) with the standard genetic code. BLASTp and BLASTx were used to search for homologous mycoviruses against the NCBI NR database. A conserved-domain database (CDD, http://www.ncbi.nlm.nih.gov/Structure/cdd/wrpsb.cgi, accessed on 7 February 2024) was queried to identify conserved motifs. CLUSTAL_X was used to perform the multiple alignment of amino acid (aa) sequences [40]. A phylogenetic analysis was conducted using the deduced aa sequence of RNA-dependent RNA polymerase (RdRp), employing the MEGA version 6.0 software [41].

### 2.7. Observation of Virions and Confirmation of FoAV1-FOM Assembly into Virus Particles

Extraction and purification of virions of FoAV1-FOM from the FOM strain T-BJ17 were performed as previously described [13]. The obtained virions were suspended in 100 µL 0.05 mol/L PBS (pH 7.2), stained with 2% uranyl acetate, and observed under a transmission electron microscope (TEM) (JEM-1230, JEOL, Tokyo, Japan). RT-PCR using FoAV1-FOM-specific primers (FoAV1-FOM-1F and FoAV1-FOM-1R, which are listed in Table S3) was employed to confirm that the extracted virions were indeed derived from FoAV1-FOM.

### 2.8. Elimination of FoAV1-FOM from Strain T-BJ17

Single-hyphal-tip culture combined with high-temperature treatment was used to eliminate FoAV1-FOM from strain T-BJ17 as in the method described previously [42]. Strain T-BJ17 was incubated on PDA plates at 25 °C for 7 d in darkness, then mycelial plugs (5 mm in diameter) were cut from the growing edge of the PDA plate and placed in the center of 20 mL water agar (WA) plates (90 mm in diameter), which were incubated at 25 °C for 3 d in darkness. Subsequently, a WA block containing hyphal tips was taken and transferred to a new PDA plate, which was incubated at 35 °C for 10 d in darkness. This process repeated until the virus was completely eliminated. RT-PCR was employed to ascertain the efficacy of the elimination of FoAV1-FOM from T-BJ17 at every third subculture.

### 2.9. Effect of FoAV1-FOM on the Phenotypes of Its Host Fungus

Mycelial plugs (5 mm in diameter) were derived from colony margins of 7-day-old cultures of T-BJ17 or T-BJ17-VF. These plugs were subsequently cultivated on PDA plates at 25 °C in the dark. Following a 6-day incubation, the diameter of the colonies of the two strains was measured. The spore concentration and the dry weight of the mycelial biomass of these two strains were determined in accordance with previously described methods [13,43].

Pathogenicity assessment of the two strains, T-BJ17 and T-BJ17-VF, was conducted on three-leaf-stage muskmelon seedlings (cv. Jingmi 5) using a slightly modified version of the methods previously described [11,44,45] by root-soaking with spore suspensions adjusted to a concentration of 1 × 10^6^ spores/mL. Briefly, muskmelon seeds were surface-sterilized with 1.5% sodium hypochlorite for 3 min and rinsed three times in sterile water before sowing. Then, the sterilized seeds were planted in substrates consisting of nutritional soil and vermiculite (1:1), which were dry-heat-sterilized at 180 °C for 5 h in advance. For inoculation, the seedlings with three true leaves were removed from the substrates and washed under running tap water to remove adhering substrates and debris. After 15 min of root-soaking in the spore suspensions, the seedlings were replanted in sterilized substrates. The seedlings inoculated with sterilized water were used as a control. The disease incidence and disease index were calculated at 25 d post inoculation according to the method described, with minor modifications [45]. The stems were rated for disease severity on the following scale: 0 = no symptoms; 1 = lesion area occupies up to 1–20% of stem; 2 = lesion area occupies up to 21–40% of the stem; 3 = lesion area occupies up to 41–60% of the stem; 4= lesion area occupies up to 61–80% of the stem; 5 = lesion area occupies up to of 81–100% of the stem. Disease incidence = number of diseased seedlings/total number of seedlings × 100%. Disease index = 100 × ∑ (number of seedlings at each scale × representative value at each scale)/(total number of seedlings × the highest representative value). The assay utilized three biological replicates, with ten muskmelon seedlings per replicate, and the test was repeated three times. A *t*-test was performed to ascertain significant statistical differences between the two strains, with the following levels of significance: *, *p* < 0.05; **, *p* < 0.01; ***, *p* < 0.001; ****, *p* < 0.0001. This was performed using GraphPad Prism version 9.5.

### 2.10. Sensitivity of T-BJ17 and T-BJ17-VF to Difenoconazole and Pydiflumetofen

Sensitivity of the two strains, T-BJ17 and T-BJ17-VF, to difenoconazole and pydiflumetofen were evaluated in vitro as described in previous studies [37,43], with minor modifications. The final concentrations of difenoconazole and pydiflumetofen were 5.00, 1.00, 0.50, 0.10, and 0.05 µg/mL, and 1, 0.50, 0.10, 0.05, and 0.01 µg/mL, respectively. The median effective concentration (EC_50_) of difenoconazole and pydiflumetofen against the two strains was calculated using previously described methods [37,43]. Three replicates were used for each strain–fungicide combination, and the experiment was repeated three times. A *t*-test was performed using GraphPad Prism version 9.5 for the statistical analysis (*, *p* < 0.05; **, *p* < 0.01; ***, *p* < 0.001; ****, *p* < 0.0001).

### 2.11. Vertical Transmission Assay

The vertical transmission of FoAV1-FOM through spores was evaluated utilizing previously established methods [46]. Briefly, after a 7 d incubation, the spores of T-BJ17 were collected in sterilized ddH_2_O and dispersed on WA plates at appropriate dilutions. Twenty-four randomly selected single-spore colonies were individually transferred to, separate new PDA plates and cultured in the dark for 7 d at 25 °C, which were then used to extract total RNA for performing the RT-PCR. RT-PCR detection of FoAV1-FOM using the FoAV1-FOM-specific primers (FoAV1-FOM-1F and FoAV1-FOM-1R, which are listed in Table S3) was conducted to determine if FoAV1-FOM was vertically transmitted via spores.

## 3. Results

### 3.1. Metatranscriptomic Identification of Mycoviruses in FOM

On the basis of the library of 148 strains of FOM, 10.55 G raw bases were obtained by metatranscriptome sequencing and deposited in the NCBI Sequence Read Archive database under the BioProject number PRJNA1039149. These reads were de novo assembled into large contigs using the scaffolding contig algorithm; 43,792 contigs were obtained from these 148 strains of FOM. With the exception of those contigs associated with the host genome, 11 contigs of over 500 bp in length that were regarded as potential viral sequences were obtained and subjected to annotation. The best matches for these 11 contigs found in the GenBank NR database are listed in Table S4. An RT-PCR was conducted to verify the presence of potential viral sequences among the tested 148 strains of FOM using the specific primers designed based on the assembled contigs (Table S2), and only three contigs (Contig6143, Contig3934, and Contig1811) were detected in T-BJ17. Sequence analysis revealed that Contig6143, Contig3934, and Contig1811 had high aa identity with the proteins encoded by dsRNA1, dsRNA2, and dsRNA3 of FoAV1, respectively, suggesting that these three contigs might compose one mycovirus.

### 3.2. Complete Sequence, Phylogenetic Analysis, and Observation of Virions of FoAV1-FOM

**Figure 1:**
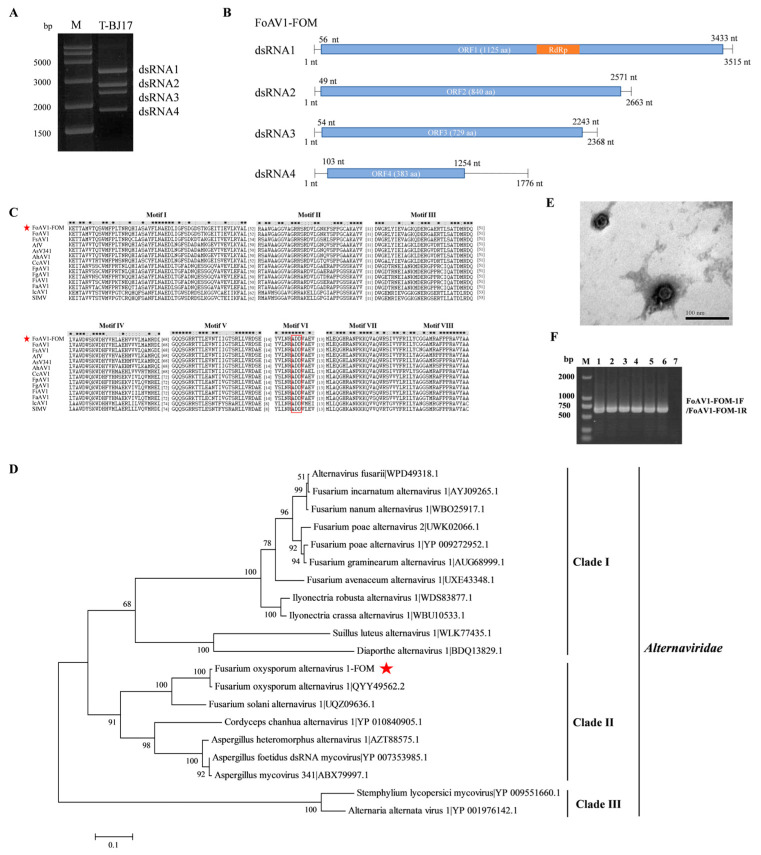
Characterization of Fusarium oxysporum alternavirus 1-FOM (FoAV1-FOM) in the *Fusarium oxysporum* f. sp. *melonis* strain T-BJ17. (**A**) Gel electrophoretic profiles of double-stranded RNA (dsRNA) extracted from strain T-BJ17, which was treated with DNase I and S1 Nuclease. M: DNA molecular marker, 250 bp DNA Ladder. (**B**) Schematic diagram of the genomic organization of FoAV1-FOM. Open reading frames (ORFs) and untranslated regions (UTRs) are indicated by blue open bars and single black lines, respectively. The yellow bar indicates the conserved RNA-dependent RNA polymerase (RdRp) domain. (**C**) Multiple alignment of amino acid (aa) sequences of the RdRp of FoAV1-FOM and twelve reference alternaviruses. Eight conserved motifs (motif I to motif VIII) were identified in the RdRp of FoAV1-FOM and the twelve reference alternaviruses. The red star indicates the position of FoAV1-FOM. The red box indicates the highly conserved ADD tripeptide. The name and GenBank accession number of these twelve reference alternaviruses are listed in Table S5. (**D**) Phylogenetic tree constructed based on the deduced aa sequence of the putative RdRp of FoAV1-FOM and nineteen reference alternaviruses using the maximum-likelihood (ML) method with 1000 bootstrap replicates. The bar scale represents a genetic distance of 0.1 aa substitutions per site. The red star indicates the position of FoAV1-FOM. The name and GenBank accession number of these nineteen reference alternaviruses are listed in Table S6. (**E**) Transmission electron microscope (TEM) image of virions of FoAV1-FOM. Scale bar = 100 nm. (**F**) Validation of FoAV1-FOM as being responsible for the formation of the virus particles extracted from strain T-BJ17. M: DNA molecular marker, DL 2000. Lane 1 to Lane 6: Products amplified by reverse transcription–polymerase chain reaction (RT-PCR) using FoAV1-FOM-specific primers (FoAV1-FOM-1F and FoAV1-FOM-1R, which are listed in Table S3) and visualized using 1% (*w*/*v*) agarose gel electrophoresis to confirm that FoAV1-FOM contributes to the assembly of virions; total RNA extracted from the virus particles was used as the RNA template. Lane 7: Blank control. Products amplified by RT-PCR using FoAV1-FOM-specific primers (FoAV1-FOM-1F and FoAV1-FOM-1R, which are listed in Table S3) and visualized using 1% (*w*/*v*) agarose gel electrophoresis; ddH_2_O was used as the template.

The dsRNA extracted from the FOM strain T-BJ17 was electrophoresed through a 1.0% (*w*/*v*) agarose gel, and four bands (of about 1.5–3.5 kb), namely dsRNA1, dsRNA2, dsRNA3, and dsRNA4, were observed (Figure 1A). The nucleotide sequences of the four dsRNAs were determined to be 3515 bp (dsRNA1), with a G + C content of 54.71%; 2663 bp (dsRNA2), with a G + C content of 56.10%; 2368 bp (dsRNA3), with a G + C content of 56.04%; and 1776 bp (dsRNA4), with a G + C content of 58.11%, respectively. The genome sequences were deposited in GenBank under the accession numbers PP482553, PP482554, PP482555, and PP482556. ORF1 in dsRNA1 was expected to encode a polypeptide of 1125 aa residues containing a conserved domain of RdRp and was 99.02% identical to its counterpart in FoAV1 (Figure 1B). ORF2 in dsRNA2 encoded an 840 aa polypeptide with 95% identity to the hypothetical protein encoded by ORF2 in FoAV1 (Figure 1B). ORF3 in dsRNA3 encoded a 729 aa polypeptide with 97.39% identity to the hypothetical protein encoded by ORF3 in FoAV1 (Figure 1B). ORF4 in dsRNA4 encoded a 383 aa polypeptide with 88.92% identity to the hypothetical protein encoded by ORF4 in FoAV1 (Figure 1B).

Multiple alignment of aa sequences of RdRp of FoAV1-FOM and twelve reference alternaviruses were conducted, and eight conserved motifs (motif I to motif VIII) were revealed in the RdRp domain. Moreover, an ADD tripeptide was found in motif VI, which is a typical characteristic of RdRp in alternaviruses (Figure 1C). Phylogenetic analysis based on the aa sequence of RdRp of FoAV1-FOM and nineteen reference alternaviruses revealed that all the twenty alternaviruses clustered into three clades (clades I, II, and III), and most alternaviruses were clustered into clade I. However, FoAV1-FOM, together with six alternaviruses, was clustered into clade II, and it was the most closely related to FoAV1 in the same subclade (Figure 1D).

Ultracentrifugation using stepwise sucrose density gradients was used to extract the possible viral particles from strain T-BJ17, and isometric particles with an average diameter of 33 nm were observed (Figure 1E). Their morphology and virion size were identical to the virions of Alternaria alternata virus 1 (AaV1), the first alternavirus that was isolated from *A. alternata* [47]. RT-PCR products were amplified from the total RNA of the virions using FoAV1-FOM-specific primers (FoAV1-FOM-1F and FoAV1-FOM-1R, which are listed in Table S3) and electrophoresed through a 1.0% (*w*/*v*) agarose gel, and the results of electrophoresis verified that FoAV1-FOM was responsible for assembling the virions extracted from the FOM strain T-BJ17 (Figure 1F). Collectively, the results mentioned above suggest that FoAV1-FOM is a novel strain of FoAV1.

### 3.3. Effect of FoAV1-FOM on the Phenotypes of Its Host

**Figure 2:**
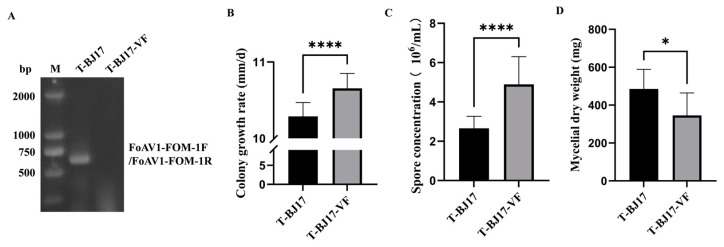
Confirmation of successful elimination of Fusarium oxysporum alternavirus 1-FOM (FoAV1-FOM) and the colony diameter, spore concentration, and dry weight of the mycelial biomass of two strains of *Fusarium oxysporum* f. sp. *melonis*, T-BJ17 and T-BJ17-VF. (**A**) Products amplified by reverse transcription–polymerase chain reaction (RT-PCR) analysis using FoAV1-FOM-specific primers (FoAV1-FOM-1F and FoAV1-FOM-1R, which are listed in Table S3) and visualized using 1% agarose gel electrophoresis to confirm the presence of FoAV1-FOM in strain T-BJ17 and the absence of FoAV1-FOM in T-BJ17-VF. (**B**) Colony diameter of the two strains, T-BJ17 and T-BJ17-VF, cultured on potato dextrose agar (PDA) plates at 25 °C for 6 d in darkness. (**C**) Spore concentration of the two strains, T-BJ17 and T-BJ17-VF, cultured on PDA plates at 25 °C for 7 d in the dark. (**D**) Average dry weight of mycelial biomass generated by the two strains, T-BJ17 and T-BJ17-VF, cultured in potato dextrose broth (PDB) for 7 d at 25 °C in darkness. Stars indicate different levels of significant differences between the two strains as determined by the *t*-test using GraphPad Prism version 9.5 software (*, *p* < 0.05; ****, *p* < 0.0001).

Strain T-BJ17-VF was obtained using single-hyphal-tip culture combined with high-temperature treatment to eliminate FoAV1-FOM from strain T-BJ17 successfully (Figure 2A), which was demonstrated to be FoAV1-FOM-free by RT-PCR analysis using FoAV1-FOM-specific primers (FoAV1-FOM-1F and FoAV1-FOM-1R, which are listed in Table S3).

The average colony growth rate of strain T-BJ17 (10.29 mm/d) was significantly lower than that of strain T-BJ17-VF (10.66 mm/d) (Figure 2B). Moreover, the average spore concentration of strain T-BJ17 (2.66 × 10^6^ spores/mL) was significantly lower than that of strain T-BJ17-VF (4.90 × 10^6^ spores/mL) (Figure 2C). However, the average dry weight of the mycelial biomass generated by strain T-BJ17 (485.6 mg) was higher than that of the mycelial biomass generated by strain T-BJ17-VF (345.6 mg) (Figure 2D and Figure S1).

**Figure 3:**
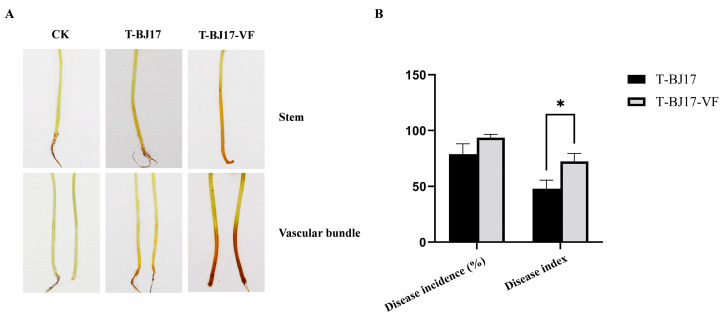
Virulence of two strains of *Fusarium oxysporum* f. sp. *melonis*, T-BJ17 and T-BJ17-VF, on muskmelon seedlings. (**A**) Disease symptoms in muskmelon seedlings inoculated with the two strains, T-BJ17 and T-BJ17-VF, at 25 d post inoculation. (**B**) Disease incidence and disease index in muskmelon seedlings inoculated with the two strains, T-BJ17 and T-BJ17-VF. Stars indicate different levels of significant differences between the two strains as determined by the *t*-test using GraphPad Prism version 9.5 software (*, *p* < 0.05).

Brown necrotic lesions at the base of the stems and vascular discoloration were observed on the muskmelon seedlings inoculated with strain T-BJ17 or strain T-BJ17-VF, which were the typical symptoms of Fusarium wilt (Figure 3A). Statistical analysis indicated that the disease incidence of muskmelon seedlings inoculated with strain T-BJ17 (78.79%) was not significantly different from that of muskmelon seedlings inoculated with strain T-BJ17-VF (93.72%) (Figure 3B). However, the disease index of muskmelon seedlings inoculated with strain T-BJ17 (47.88) was much lower than that of muskmelon seedlings inoculated with strain T-BJ17-VF (72.47) (Figure 3B). Thus, it was concluded that the infection of FoAV1-FOM could confer hypovirulence on its host fungus.

**Figure 4:**
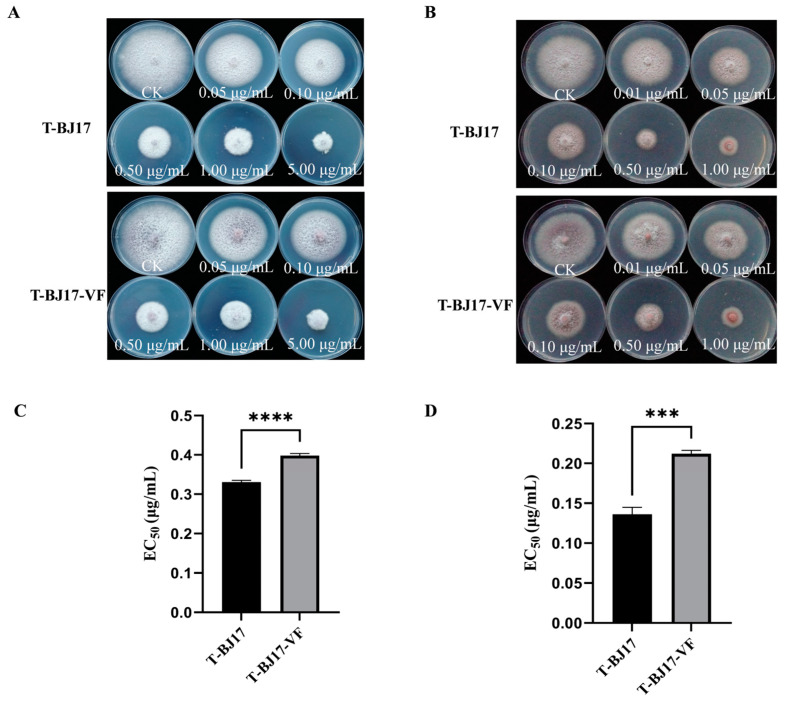
Sensitivity of two strains of *Fusarium oxysporum* f. sp. *melonis*, T-BJ17 and T-BJ17-VF, to difenoconazole and pydiflumetofen. (**A**) Effect of difenoconazole on colony growth of the two strains, T-BJ17 and T-BJ17-VF. (**B**) Effect of pydiflumetofen on colony growth of the two strains, T-BJ17 and T-BJ17-VF. (**C**) Median effective concentration (EC_50_) of difenoconazole against the two strains, T-BJ17 and T-BJ17-VF. (**D**) EC_50_ of pydiflumetofen against the two strains, T-BJ17 and T-BJ17-VF. Stars indicate different levels of significant differences between the two strains as determined by the *t*-test using GraphPad Prism version 9.5 software (***, *p* < 0.001; ****, *p* < 0.0001).

Difenoconazole and pydiflumetofen inhibited colony growth of the two strains, T-BJ17 and T-BJ17-VF (Figure 4A,B); however, the EC_50_ values of difenoconazole and pydiflumetofen against strain T-BJ17 (0.3308 μg/mL and 0.1362 μg/mL, respectively) were significantly lower than those of difenoconazole and pydiflumetofen against strain T-BJ17-VF (0.3981 μg/mL and 0.2121 μg/mL, respectively) (Figure 4C,D). These data indicated that FoAV1-FOM infection increased the sensitivity of strain T-BJ17 to difenoconazole and pydiflumetofen.

### 3.4. Vertical Transmission of FoAV1-FOM

**Figure 5:**
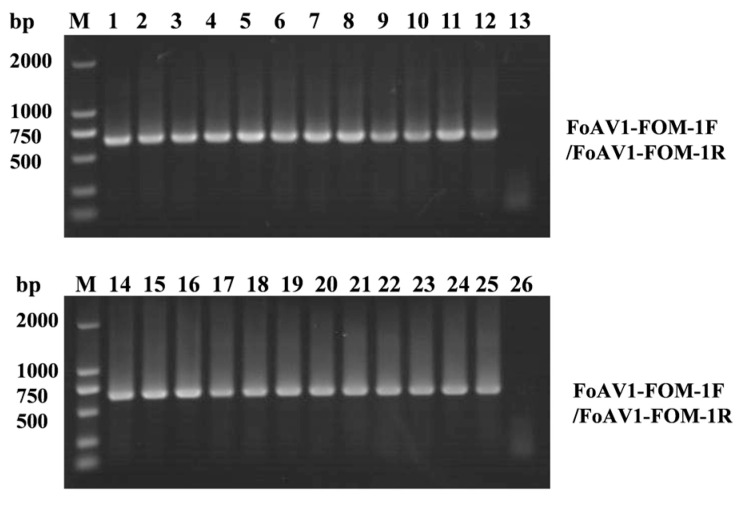
Validation of the presence of Fusarium oxysporum alternavirus 1-FOM (FoAV1-FOM) in the 24 single-spore cultures derived from strain T-BJ17. M: DNA molecular marker, DL 2000. Lane 1 to Lane 12 and Lane 14 to Lane 25: Products amplified by reverse transcription–polymerase chain reaction (RT-PCR) using FoAV1-FOM-specific primers (FoAV1-FOM-1F and FoAV1-FOM-1R, which are listed in Table S3) and electrophoresed through a 1.0% (*w*/*v*) agarose gel, in which total RNA extracted from the 24 single-spore cultures derived from *Fusarium oxysporum* f. sp. *melonis* strain T-BJ17 was used as the RNA template. Lane 13 and Lane 26: Negative controls. Products amplified by RT-PCR using FoAV1-FOM-specific primers (FoAV1-FOM-1F and FoAV1-FOM-1R, which are listed in Table S3) and electrophoresed through a 1.0% (*w*/*v*) agarose gel, in which total RNA extracted from the two single-spore cultures derived from *F. oxysporum* f. sp. *melonis* strain T-BJ17-VF was used as the RNA template.

RT-PCR was conducted using FoAV1-FOM-specific primers (FoAV1-FOM-1F and FoAV1-FOM-1R, which are listed in Table S3), and the RT-PCR products were electrophoresed through a 1.0% (*w*/*v*) agarose gel, in which total RNA extracted from the 24 single-spore cultures derived from FOM strain T-BJ17 was used as the RNA template. As a result, FoAV1-FOM could be detected in these 24 single-spore cultures, indicating that FoAV1-FOM could achieve 100% vertical transmission via spores (Figure 5).

## 4. Discussion

Metatranscriptome sequencing technology is widely used, which is dramatically expanding the understanding of the diversity of mycoviruses and increasing the discovery of novel mycoviruses in plant pathogenic fungi. In the present study, a survey of mycoviruses was conducted in 148 strains of FOM by metatranscriptome sequencing, and a novel strain of FoAV1 was isolated from T-BJ17 and designated as FoAV1-FOM, which was the causal agent of muskmelon Fusarium wilt. FoAV1, an alternavirus, was identified in *F. oxysporum* isolated from diseased stems of *L. brownii* with the typical symptoms of Fusarium wilt [11]. Besides FoAV1, there were seven other alternaviruses determined in seven species of *Fusarium*, respectively [11,13,14,15,16,17,18,19]. However, to the best of our knowledge, this is the first report of an alternavirus infecting FOM and causing muskmelon Fusarium wilt.

It was stated that the criteria for demarcating species should consider the number of dsRNA segments and the presence and homology of dsRNA 4 (https://ictv.global/files/proposals/approved?fid=11959#block-teamplus-page-title, accessed on 5 March 2024). Thus far, eighteen mycoviruses have been assigned as members of the family *Alternaviridae* [11,13,14,15,16,17,18,19,47,48,49,50,51,52,53,54,55]. Except for FaAV1, which is composed of two dsRNA segments [13], the remaining seventeen alternaviruses contain three or four dsRNA segments [11,14,15,16,17,18,19,47,48,49,50,51,52,53,54,55]. Among them, eleven alternaviruses, namely Aspergillus heteromorphus alternavirus 1 (AheAV1) [49], Cordyceps chanhua alternavirus 1 (CcAV1) [52], Dactylonectria torresensis alternavirus 1 (DtAV1) [53], FgAV1 [14], FiAV1 [15], FnAV1 [16], FpgAV1 [18], FpAV1 [17], Ilyonectria crassa alternavirus 1 (IcAV1) [51], Ilyonectria robusta alternavirus 1 (IrAV1) [53], and Suillus luteus alternavirus 1 (SluAV1) [55], contain three dsRNA segments, while six alternaviruses, including AaV1 [47], Aspergillus foetidus virus (AfV) [54], Aspergillus virus 341 (AsV341) [50], FoAV1 [11], FsAV1 [19], and Stemphylium lycopersici alternavirus 1 (SlAV1) [48], contain four dsRNA segments. Usually, dsRNA 1 encodes RdRp, dsRNA 2 encodes a conserved protein with an unknown function, dsRNA 3 encodes capsid protein (CP), and dsRNA 4, when present, encodes a non-conserved protein with an unknown function that is often non-homologous among alternaviruses. In the current study, FoAV1-FOM contained tetrapartite dsRNA genomes, and dsRNA1, dsRNA2, and dsRNA3 of FoAV1-FOM shared high sequence identity (95%-99.02%) with counterparts of FoAV1, while the sequence identity of dsRNA4 between FoAV1 and FoAV1-FOM was slightly lower (88.92%).

Only three (FoAV1, SlAV1, and FaAV1) of the eighteen alternaviruses were reported to alter the biological characteristics of their fungal hosts or recipient strains [11,13,48]. The FoAV1 isolated from *F. oxysporum*, which causes Fusarium wilt of *L. brownii*, did not alter the colony morphology but decreased the virulence of *F. oxysporum* f. sp. *momordicae*, a recipient strain of FoAV1 that causes Fusarium wilt of bitter gourd [11]. SlAV1 mediated hypovirulence by reducing pigmentation and suppressing the production of Altersolanol A in its host [48]. However, the infection of FaAV1 increased the mycelial biomass and conferred hypervirulence on its host fungus [13]. In this study, FoAV1-FOM infection induced hypovirulence as well as the promotion of mycelial biomass, and it decreased the colony growth and spore production of its host.

Mycoviruses utilize sexual (basidiospores and ascospores) or asexual spores (conidiophores) for their vertical transmission to the next generation, especially the asexual spores (conidiophores), which are most frequently used by mycoviruses for transmitting to progeny cells [56]. The transmission rate varies among different mycoviruses andd fungal isolates and depends on the age of the fungal colonies [46,56,57,58]. Two mitoviruses, Fusarium verticillioides mitovirus 1 (FvMV1) and Fusarium andiyazi mitovirus 1 strain 162 (FaMV1-162), exhibited a 100% vertical transmission rate via spores, which infected *F. verticillioides* and *F. andiyazi*, respectively [46]. One chrysovirus, Fusarium oxysporum f. sp. dianthi virus 1 (FodV1), associated with *F. oxysporum* f. sp. *dianthi*, was capable of 100% vertical transmission via spores [5]. In addition, a botybirnavirus, Alternaria alternata botybirnavirus 1-AT1 (AaBRV1-AT1), identified in the *A. tenuissima* strain TJ-NH-51S-4, could achieve 100% vertically transmission via asexual spores [42]. In the current study, FoAV1-FOM, like FvMV1, FaMV1-162, FodV1, and AaBRV1-AT1, could also achieve 100% vertical transmission via spores.

In our previous studies, the infection of Alternaria alternata chrysovirus 1-AT1 (AaCV1-AT1) or AaBRV1-AT1 decreased the sensitivity of their hosts to difenoconazole [42,59]. However, in the present study, FoAV1-FOM infection increased the sensitivity of its host FOM strain T-BJ17 to difenoconazole. There were no reports recorded that studied how infection of a mycovirus can affect the sensitivity of its host fungus to pydiflumetofen. This study demonstrated that FoAV1-FOM infection increased the sensitivity of its host fungus *F. oxysporum* f. sp. *niveum* to pydiflumetofen, and this is the first report of the increased sensitivity of the host fungus infected by a mycovirus to pydiflumetofen.

The present study identified a novel strain of FoAV1 (FoAV1-FOM) and characterized, for the first time, the hypovirulence and increased sensitivity of its host FOM fungus to difenoconazole and pydiflumetofen that is induced by FoAV1-FOM infection. Our study also established an experimental system that could be utilized to study the interaction between FoAV1-FOM and its host.

## Data Availability

The sequences reported in the present manuscript have been deposited in the GenBank database (https://www.ncbi.nlm.nih.gov/genbank/) under accession numbers PP482553, PP482554, PP482555, and PP482556. The raw data of the metatranscriptome sequencing are available from the NCBI Sequence Read Archive (https://www.ncbi.nlm.nih.gov/sra, BioProject PRJNA1039149).

## Supplementary Materials

The following are available online at https://www.mdpi.com/article/10.3390/v16060901/s1, Table S1: Information on the 148 strains of *Fusarium oxysporum* f. sp. *melonis* (FOM) used for metatranscriptomic sequencing. Table S2: Primers used to verify the presence of putative mycoviruses in the 148 strains of *Fusarium oxysporum* f. sp. *melonis* (FOM) strains in this study. Table S3: Primers used to determine the complete genome sequence of Fusarium oxysporum alternavirus 1-FOM (FoAV1-FOM) and to verify the presence of FoAV1-FOM in the *Fusarium oxysporum* f. sp. *melonis* strain T-BJ17 in this study. Table S4: Alignment information of putative mycoviruses found in 148 strains of *Fusarium oxysporum* f. sp. *melonis* (FOM) using metatranscriptome sequencing. Table S5: The information on the twelve reference alternaviruses retrieved from the GenBank database (National Center for the Biotechnology Information) and used to conduct the multiple alignment. Table S6: The information on the nineteen reference alternaviruses retrieved from the GenBank database (National Center for the Biotechnology Information) and used to conduct the phylogenetic analysis. Figure S1: Colony morphology of two strains of *Fusarium oxysporum* f. sp. *melonis*, T-BJ17 and T-BJ17-VF, cultured on potato dextrose agar (PDA) plates at 25 °C for 10 d in darkness.
