# Supplementary material for: A Novel Strain of Fusarium oxysporum Alternavirus 1 Isolated from Fusarium oxysporum f. sp. melonis Strain T-BJ17 Confers Hypovirulence and Increases the Sensitivity of Its Host Fungus to Difenoconazole and Pydiflumetofen
Source: Viruses. 2024 Jun 2;16(6):901. doi: 10.3390/v16060901 (PMC11209391; doi:10.3390/v16060901)
Supplement: Supplementary file 1 [file viruses-16-00901-s001.zip › viruses-2944500-supplementary.pdf]

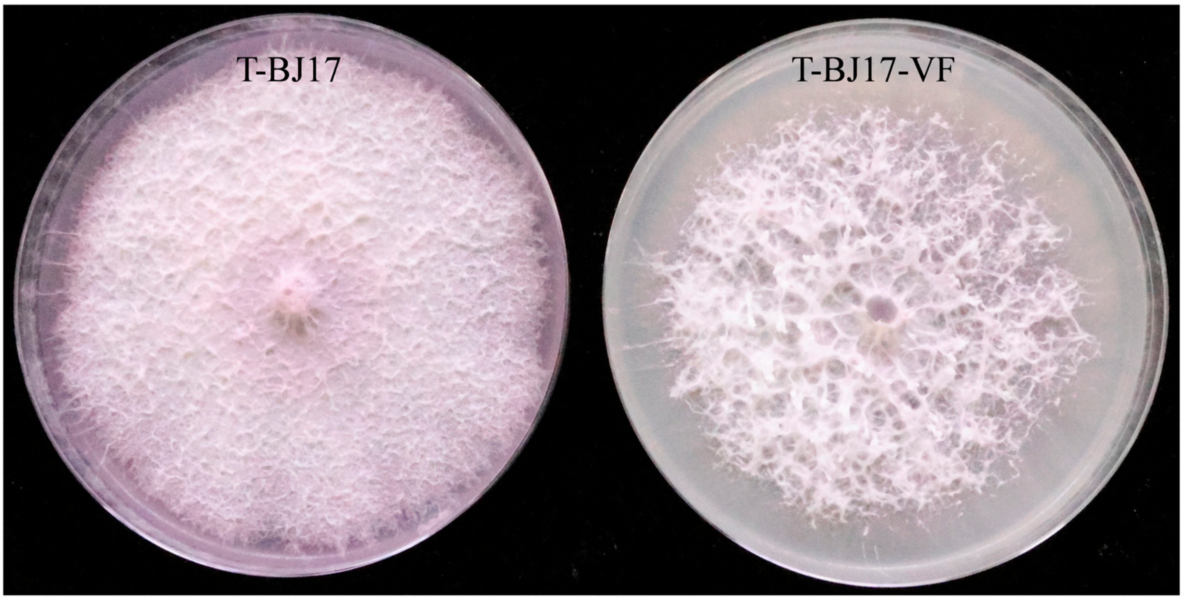

Figure S1. Colony morphology of two strains of 482 *Fusarium oxysporum* f. sp. *melonis*, T-BJ17 and T-BJ17-VF, cultured on potato dextrose agar (PDA) 483 plates at 25 °C for 10 d in darkness.

Table S1. Information of 148 strains of *Fusarium oxysporum* f. sp. *melonis* (FOM) used for meta-transcriptomic sequencing.

Group	Strain Code	Origin	Year
	T-BJ1	Shunyi district, Beijing municipality	2011
	T-BJ2	Shunyi district, Beijing municipality	2011
T1	T-BJ3	Shunyi district, Beijing municipality	2011
	T-BJ4	Shunyi district, Beijing municipality	2011
	T-BJ5	Shunyi district, Beijing municipality	2011
	T-BJ6	Shunyi district, Beijing municipality	2012
T2	T-BJ8	Shunyi district, Beijing municipality	2012
	T-BJ9	Shunyi district, Beijing municipality	2012
	T-BJ11	Daxing district, Beijing municipality	2011
	T-BJ12	Daxing district, Beijing municipality	2011
T3	T-BJ13	Daxing district, Beijing municipality	2011
	T-BJ14	Shunyi district, Beijing municipality	2011
	T-BJ15	Shunyi district, Beijing municipality	2018
	T-BJ16	Shunyi district, Beijing municipality	2018
	T-BJ17	Shunyi district, Beijing municipality	2018
T4	T-BJ18	Shunyi district, Beijing municipality	2018
	T-BJ19	Shunyi district, Beijing municipality	2018
	T-BJ20	Shunyi district, Beijing municipality	2018
	T-BJ21	Shunyi district, Beijing municipality	2018
	T-FJ1	Fuzhou city, Fujian province	2011
T5	T-FJ2	Fuzhou city, Fujian province	2011
	T-FJ3	Fuzhou city, Fujian province	2011
	T-FJ4	Fuzhou city, Fujian province	2011
	T-FJ5	Fuzhou city, Fujian province	2011
	T-FJ6	Fuzhou city, Fujian province	2011
T6	T-FJ7	Fuzhou city, Fujian province	2010
	T-FJ8	Putian city, Fujian province	2010
	T-FJ9	Fujian province	2012
	T-FJ10	Fujian province	2012
	T-FJ11	Fuzhou city, Fujian province	2012
T7	T-FJ12	Fuzhou city, Fujian province	2012
	T-FJ13	Fuzhou city, Fujian province	2012
	T-FJ14	Fuzhou city, Fujian province	2012
	T-FJ15	Fuzhou city, Fujian province	2012
T8	T-FJ16	Fuzhou city, Fujian province	2012
	T-FJ17	Fuzhou city, Fujian province	2012

	T-FJ18	Fuzhou city, Fujian province	2012
	T-FJ19	Fuzhou city, Fujian province	2012
	T-FJ20	Fuzhou city, Fujian province	2012
	T-FJ21	Fuzhou city, Fujian province	2012
T9	T-FJ22	Fuzhou city, Fujian province	2012
	T-FJ23	Fuzhou city, Fujian province	2012
	T-FJ24	Fuzhou city, Fujian province	2012
	T-GD1	Foshan city, Guangdong province	2016
	T-HLJ1	Tsitsihar city, Heilongjiang province	2011
T10	T-HLJ2	Tsitsihar city, Heilongjiang province	2011
	T-HLJ3	Tsitsihar city, Heilongjiang province	2011
	T-HLJ4	Suihua city, Heilongjiang province	2011
	T-HLJ5	Suihua city, Heilongjiang province	2011
	T-HuN1	Liuyang city, Hunan province	2016
T11	T-JL1	Jiutai city, Jilin province	2011
	T-JL2	Jiutai city, Jilin province	2011
	T-JL3	Jilin city, Jilin province	2011
	T-JX1	Nanchang city, Jiangxi province	2017
	T-JX2	Nanchang city, Jiangxi province	2017
T12	T-JX3	Nanchang city, Jiangxi province	2017
	T-JX4	Nanchang city, Jiangxi province	2017
	T-JX5	Nanchang city, Jiangxi province	2017
	T-JX6	Nanchang city, Jiangxi province	2018
	T-JX7	Nanchang city, Jiangxi province	2018
T13	T-JX8	Nanchang city, Jiangxi province	2017
	T-JX9	Nanchang city, Jiangxi province	2018
	T-JX10	Nanchang city, Jiangxi province	2018
	T-JX11	Nanchang city, Jiangxi province	2018
	T-JX12	Nanchang city, Jiangxi province	2018
T14	T-JX13	Nanchang city, Jiangxi province	2018
	T-JX14	Ganzhou city, Jiangxi province	2018
	T-JX15	Ganzhou city, Jiangxi province	2018
	T-JX16	Ganzhou city, Jiangxi province	2018
	T-JX17	Nanchang city, Jiangxi province	2018
T15	T-JX18	Nanchang city, Jiangxi province	2018
	T-JX19	Nanchang city, Jiangxi province	2018
	T-JX20	Nanchang city, Jiangxi province	2018
	T-JX21	Nanchang city, Jiangxi province	2018
	T-JX22	Nanchang city, Jiangxi province	2018
T16	T-JX23	Nanchang city, Jiangxi province	2018
	T-JX24	Nanchang city, Jiangxi province	2018
	T-JX25	Nanchang city, Jiangxi province	2018
	T-JX26	Nanchang city, Jiangxi province	2018
	T-JX27	Nanchang city, Jiangxi province	2018
T17	T-JX28	Nanchang city, Jiangxi province	2018
	T-JX29	Nanchang city, Jiangxi province	2018
	T-JX30	Nanchang city, Jiangxi province	2018
	T-JX31	Nanchang city, Jiangxi province	2018
	T-JX32	Nanchang city, Jiangxi province	2018
T18	T-LN1	Chaoyang city, Liaoning province	2011
	T-LN2	Chaoyang city, Liaoning province	2011
	T-LN3	Shenyang city, Liaoning province	2011
	T-LN4	Shenyang city, Liaoning province	2011
	T-LN5	Shenyang city, Liaoning province	2011
T19	T-LN6	Shenyang city, Liaoning province	2011
	T-LN7	Shenyang city, Liaoning province	2011
	T-LN8	Shenyang city, Liaoning province	2011
	T-LN9	Shenyang city, Liaoning province	2011
	T-LN10	Shenyang city, Liaoning province	2011
T20	T-LN11	Shenyang city, Liaoning province	2011
	T-LN12	Shenyang city, Liaoning province	2016
	T-LN13	Fushun city, Liaoning province	2016
	T-LN14	Shenyang city, Liaoning province	2016
T21	T-LN15	Fushun city, Liaoning province	2016
	T-LN16	Fushun city, Liaoning province	2016
	T-LN17	Fushun city, Liaoning province	2016

	T-LN18	Shenyang city, Liaoning province	2016
	T-LN19	Shenyang city, Liaoning province	2016
	T-LN20	Shenyang city, Liaoning province	2016
T22	T-LN21	Shenyang city, Liaoning province	2016
	T-LN22	Shenyang city, Liaoning province	2016
	T-LN23	Shenyang city, Liaoning province	2016
	T-LN24	Shenyang city, Liaoning province	2016
	T-LN25	Shenyang city, Liaoning province	2016
T23	T-LN26	Shenyang city, Liaoning province	2016
	T-NM1	Chifeng city, Inner Mongolia autonomous region	2011
	T-NM2	Chifeng city, Inner Mongolia autonomous region	2011
	T-NM3	Chifeng city, Inner Mongolia autonomous region	2011
	T-NM4	Chifeng city, Inner Mongolia autonomous region	2011
T24	T-NM5	Chifeng city, Inner Mongolia autonomous region	2011
	T-NX1	Yinchuan city, Ningxia Hui autonomous region	2018
	T-NX2	Yinchuan city, Ningxia Hui autonomous region	2018
	T-NX3	Yinchuan city, Ningxia Hui autonomous region	2018
	T-NX4	Yinchuan city, Ningxia Hui autonomous region	2018
T25	T-NX5	Yinchuan city, Ningxia Hui autonomous region	2018
	T-NX6	Yinchuan city, Ningxia Hui autonomous region	2018
	T-NX7	Yinchuan city, Ningxia Hui autonomous region	2018
	T-NX8	Yinchuan city, Ningxia Hui autonomous region	2018
	T-NX9	Yinchuan city, Ningxia Hui autonomous region	2018
T26	T-NX10	Yinchuan city, Ningxia Hui autonomous region	2018
	T-SD1	Xintai city, Shandong province	2016
	T-SD2	Xintai city, Shandong province	2016
	T-SD3	Xintai city, Shandong province	2016
	T-SD4	Xintai city, Shandong province	2016
T27	T-SD5	Xintai city, Shandong province	2016
	T-SD6	Xintai city, Shandong province	2016
	T-ZJ1	Yiwu city, Zhejiang province	2016
T2	T-ZJ2	Yiwu city, Zhejiang province	2016
	T-ZJ3	Yiwu city, Zhejiang province	2016
	FJAT-9062	Fuzhou city, Fujian province	2010
T28	FJAT-9230	Putian city, Fujian province	2010
	FJAT-31372	Fuzhou city, Fujian province	2017
	FJAT-31376	Fuzhou city, Fujian province	2017
	FJAT-129	Fuzhou city, Fujian province	2003
	FJAT-130	Fuzhou city, Fujian province	2003
T29	FJAT-9172	Fuzhou city, Fujian province	2010
	FJAT-9173	Fuzhou city, Fujian province	2010
	FJAT-9232	Putian city, Fujian province	2010
	FJAT-9233	Putian city, Fujian province	2010
T30	FJAT-31375	Fuzhou city, Fujian province	2017
	FJAT-31378	Fuzhou city, Fujian province	2017
	HJY	Beijing municipality	2018

Table S2. Primers used to verify the presence of putative mycoviruses in 148 strains of *Fusarium oxysporum* f. sp. *melonis* (FOM) in this study.

Primer Name	Sequence (5'-3')
contig 318-1F	TACCCAACACAACTCTCTTCCTCT
contig318-1R	GTTTCCACTTCTCTGTATCGTCAA
contig1322-1F	GGGAAGGTAGTTTTGGAACACACACA
contig1322-1R	GTAATACTCAACATCATCAGGCACGG
contig 1321-1F	CATTGCTTCCGATGCTTCAAAAACTC
contig 1321-1R	GCACTCACGACAAATACATATGCCCG
contig 291-1F	AGTGTGCATGACGATCGGTTTCTT
contig 291-1R	GCCTTTTGTGTTCTTGTTCTGGGT
contig 489-1F	TCCCATTAGTCTGATTAGTTCTGTTC
contig 489-1R	TCGTAAGCTTTCTTCGATACTCTCGA
contig 44-1F	ATCTGAGTGGTCGCTCCTGATTTCGG
contig 44-1R	TGACGGTGTACAACGGTCCATTGCCT
contig 13-1F	TATGTCCAAAGCACCCAAACCCCCCG
contig 13-1R	GGCTACACCAAGACCGTCACCCGCAG

contig 4844-1F	CTGCTGTGGGTGATGTTGAGGTAGTG
contig 4844-1R	ATCCTTTGTTTTAATGATGGGGTTGC
contig 6143-1F	TAGGTCGTGGACGGTTTGATGAGG
contig 6143-1R	CCAAGAGGCAGGAAGCAATTTGTG
contig 3934-1F	TTCGCCTCTCAAAATCCAACCTTC
contig 3934-1R	GTATCAAACACAGCCCCCCCCCAG
contig 1811-1F	AATACCCATCTCAGACCGCC
contig 1811-1R	TGTTCATTATGTCCCTTTCGC

Table S3. Primers used to determine the complete genome sequence of Fusarium oxysporum alternavirus 1-FOM (FoAV1-FOM) and verify the presence of FoAV1-FOM in *Fusarium oxysporum* f. sp. *melonis* (FOM) strain T-BJ17 in this study.

Primer Name	Sequence (5'-3')
RACE3	CGATCGATCATGATGCAATGC
RACE3RT	CGATCGATCATGATGCAATGCNNNNNN
PC3-T7 Loop adapter	p-GGATCCCGGGAATTCGGTAATACGACTCA<br>CTATATTTTTATAGTGAGTCGTATTA-OH
PC2	CCGAATTCCCGGGATCC
dsRNA1-3end-A	AGGATGGGTCAGGTGATGTA
dsRNA1-3end-B	GTGGACGGTTTGATGAGGA
dsRNA1-5end-A	CCAGGGAACGGACCAAAC
dsRNA1-5end-B	GAAAGCACATACTGGCATAGC
dsRNA2-3end-A	CCCGCACTACGCTTCACA
dsRNA2-3end-B	GGTCGCTTCTTACTCTGTTGA
dsRNA2-5end-A	ATCACCACCCACAAAGACCC
dsRNA2-5end-B	GCCAGACTCGGCTCGGGAGA
dsRNA3-3end-A	TCCTGGCTGGTCGTTCTA
dsRNA3-3end-B	CCGCACCTTTCGTAACAC
dsRNA3-5end-A	TCATCCGAGGTCAACTGC
dsRNA3-5end-B	CGGCAGAGGGCTTTGGAT
dsRNA4-3end-A	GTTCTATTGTCGGCGTCGT
dsRNA4-3end-B	GTATGCGTGCCGTGGTC
dsRNA4-5end-A	GCTAAGACATCGGCAGGGAG
dsRNA4-5end-B	CGCCCTCATAACGGCAGAC
FoAV1-FOM-1F	TAGGTCGTGGACGGTTTGATGAGG
FoAV1-FOM-1R	CCAAGAGGCAGGAAGCAATTTGTG

Table S4. Alignment information of putative mycoviruses found in 148 strains of *Fusarium oxysporum* f. sp. *melonis* (FOM) using metatranscriptome sequencing.

No.	Contig ID	Length (Base Pair)	Best Match	Query Cover	Identity	E-value
1	Contig6143	3483	Fusarium oxysporum alternavirus 1	79%	99.02%	0
2	Contig3934	2749	Fusarium oxysporum alternavirus 1	91%	95.00%	0
3	Contig1811	2323	Fusarium oxysporum alternavirus 1	93%	97.38%	0
4	First_Contig13	2786	Fusarium sacchari chrysovirus 1	94%	98.97%	0
5	First_Contig44	2708	Fusarium sacchari chrysovirus 1	91%	99.40%	0
6	First_Contig291	2572	Fusarium sacchari chrysovirus 1	97%	99.40%	0
7	Contig1321	3513	Fusarium sacchari chrysovirus 1	97%	99.91%	0
8	Contig1322	3531	Fusarium sacchari chrysovirus 1	96%	97.54%	0
9	Contig318	2683	Fusarium oxysporum ourmia-like virus	78%	90.31%	0
10	Contig489	2260	Fusarium asiaticum mitovirus 5	90%	82.53%	0
11	Contig4844	7206	Cladosporium cladosporioides negative-stranded RNA virus 2	96%	47.95%	0

Table S5. The information of twelve reference alternaviruses retrieved from GenBank database (National Center for the Biotechnology Information) and used to conduct multiple alignment.

Reference Virus	GenBank Accession Number
Aspergillus foetidus dsRNA mycovirus (AfV)	YP_007353985.1
Aspergillus heteromorphus alternavirus 1 (AhAV1)	AZT88575.1

Aspergillus mycovirus 341 (AsV341)	ABX79997.1
Cordyceps chanhua alternavirus 1 (CcAV1)	UPH33984.1
Fusarium avenaceum alternavirus 1 (FaAV1)	UXE43348.1
Fusarium graminearum alternavirus 1 (FgAV1)	YP_009449439.1
Fusarium incarnatum alternavirus 1 (FiAV1)	AYJ09265.1
Fusarium oxysporum alternavirus 1 (FoAV1)	QYY49562.2
Fusarium poae alternavirus 1 (FpAV1)	YP_009272952.1
Fusarium solani alternavirus 1 (FsAV1)	UQZ09636.1
Ilyonectria crassa alternavirus 1 (IcAV1)	WBU10533.1
Stemphylium lycopersici mycovirus (SlmV)	YP_009551660.1

Table S6. The information of nineteen reference alternaviruses retrieved from GenBank database (National Center for the Biotechnology Information) and used to conduct phylogenetic analysis.

Reference Virus	GenBank Accession Number
Alternaria alternata virus 1	YP_001976142.1
Alternavirus fusarii	WPD49318.1
Aspergillus foetidus dsRNA mycovirus	YP_007353985.1
Aspergillus heteromorphus alternavirus 1	AZT88575.1
Aspergillus mycovirus 341	ABX79997.1
Cordyceps chanhua alternavirus 1	YP_010840905.1
Diaporthe alternavirus 1	BDQ13829.1
Fusarium avenaceum alternavirus 1	UXE43348.1
Fusarium graminearum alternavirus 1	AUG68999.1
Fusarium incarnatum alternavirus 1	AYJ09265.1
Fusarium nanum alternavirus 1	WBO25917.1
Fusarium oxysporum alternavirus 1	QYY49562.2
Fusarium poae alternavirus 1	YP_009272952.1
Fusarium poae alternavirus 2	UWK02066.1
Fusarium solani alternavirus 1	UQZ09636.1
Ilyonectria crassa alternavirus 1	WBU10533.1
Ilyonectria robusta alternavirus 1	WDS83877.1
Stemphylium lycopersici mycovirus	YP_009551660.1
Suillus luteus alternavirus 1	WLK77435.1
